# Supplementary material for: Lysates of Methylococcus capsulatus Bath induce a lean-like microbiota, intestinal FoxP3+RORγt+IL-17+ Tregs and improve metabolism
Source: Nat Commun. 2021 Feb 17;12:1093. doi: 10.1038/s41467-021-21408-9 (PMC7889900; doi:10.1038/s41467-021-21408-9)
Supplement: Supplementary file 1 — Supplementary Information [file 41467_2021_21408_MOESM1_ESM.pdf]

Supplementary Figure 1

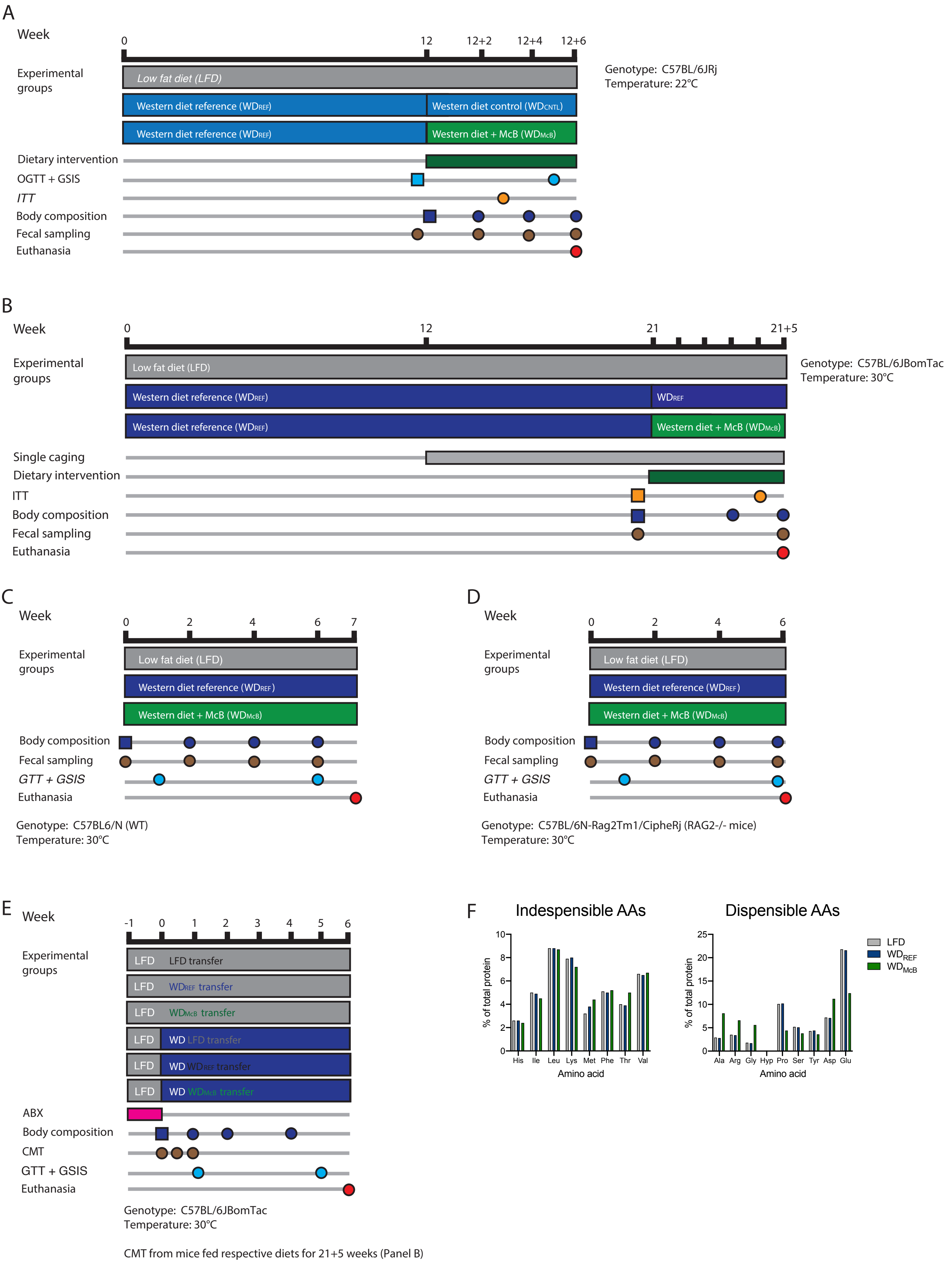

Supplementary Figure 1: Experimental layouts and diet amino acid composition. A) Study outline of two independent experiments with initial Western diet (WD_{REF}) feeding for 12 weeks at 22°C and oral glucose tolerance test (OGTT), glucose-stimulated insulin secretion (GSIS), and body composition by MR scan assessed after 11 weeks to stratify groups followed by dietary intervention for 6 weeks of feeding either WD including McB lysate (WD_{McB}) or a matched WD control diet (WD_{CNTL}). During the experimental period body composition, OGTT, GSIS, and fresh fecal sampling were carried out at the indicated intervals. An insulin tolerance test (ITT) was carried out in the first of two experiments at the indicated timepoint. The second experiment included a reference group fed low fat diet (LFD) fed mice. Groups or procedures featured uniquely in one experiment are italicized. **B)** Study outline with initial Western diet (WD_{REF}) feeding for 21 weeks at 30°C. Insulin tolerance test (ITT) and body composition by MR scan were assessed after 20 weeks to stratify groups followed by dietary intervention for 5 weeks of feeding with either WD_{REF} or WD including McB lysate (WD_{McB}). A reference group fed low fat diet (LFD) was included throughout the experiment (week 0 to week 21+5). Body composition, ITT, and fresh fecal sampling were carried out at the indicated intervals. Mice were co-housed the first 12 weeks and then separated to individual cages. **C-D)** Study outline of 'weight-matched' WT mice and $RAG2^{-/-}$ mice fed experimental diets for 7 and 6 weeks, respectively, at 30°C. The two studies were run in parallel and followed the same scheme with the exception of OGTT + GSIS (italic letters), which was performed in $RAG2^{-/-}$ only. Body composition, OGTT + GSIS, and fresh fecal sampling were carried out at the indicated intervals. **E)** Study outline of cecal microbiota transfer (CMT) studies at 30°C. Mice were acclimatized on LFD and remained on this during 7 days of ABX treatment. Then, 50% of the mice were shifted to WD_{REF}, while the other half remained on LFD for the following 5 weeks of intervention. Body composition, CMT and OGTT + GSIS were carried out at the indicated time intervals. **F)** Relative distribution of indispensable amino acids (left panel) and dispensable amino acids (right panel) in the experimental diets.

Supplementary Figure 2

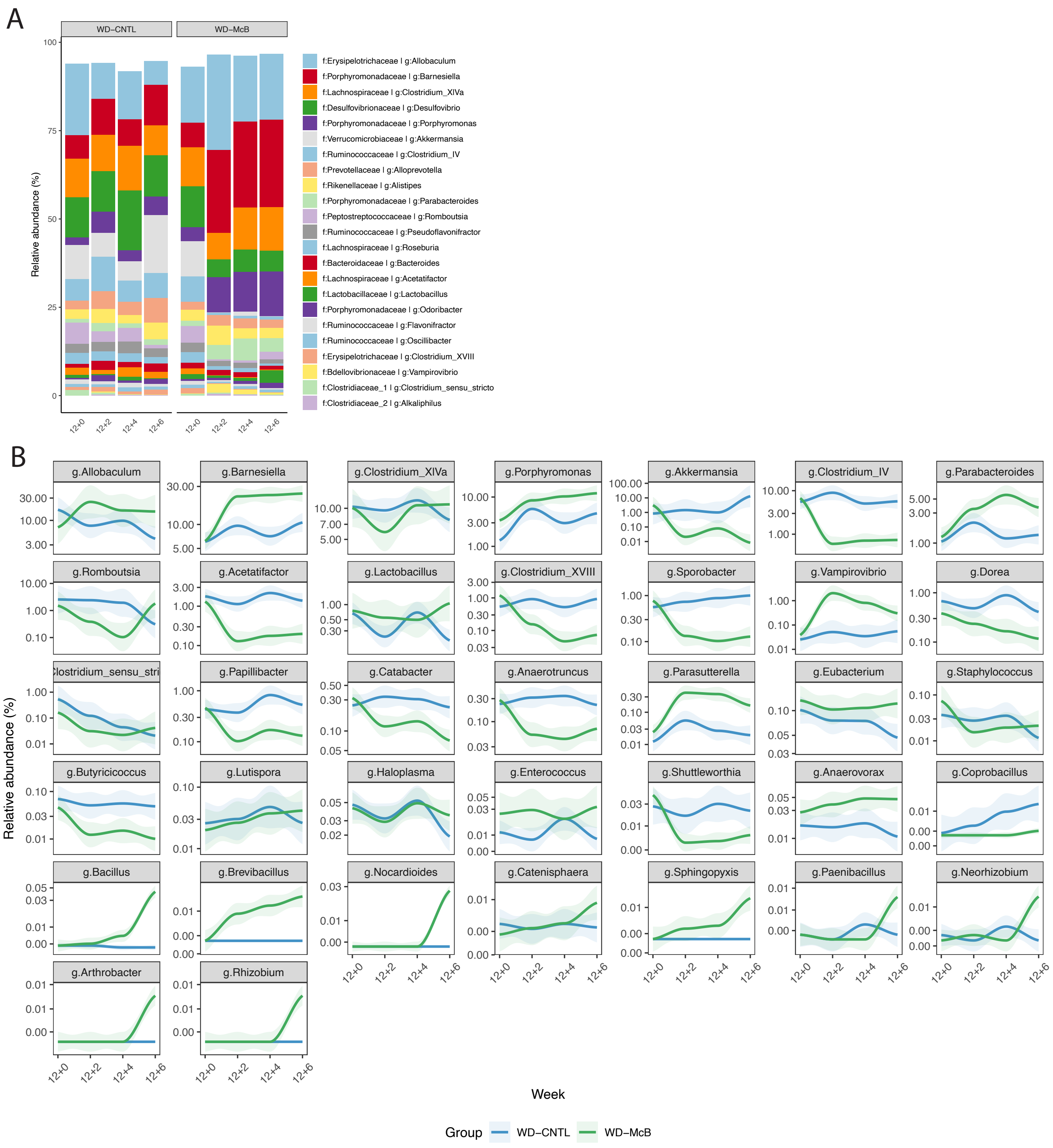

Supplementary Figure 2: Gut microbiota abundance post dietary intervention. A) Taxasummary of most abundant bacterial genera showing mean relative abundance in percentage of the indicated family and genera in each group at indicated time point. **B)** Deseq analysis of fecal bacterial genera abundances significantly regulated by McB intervention compared to the WD_{CNTL} (p.adj. < 0.05). Relative abundance in % in each group and variation are shown for each regulated genus at the sampled time points. Fold-change and adjusted p values of individual are indicated in Supplementary Table 3.

Supplementary Figure 3

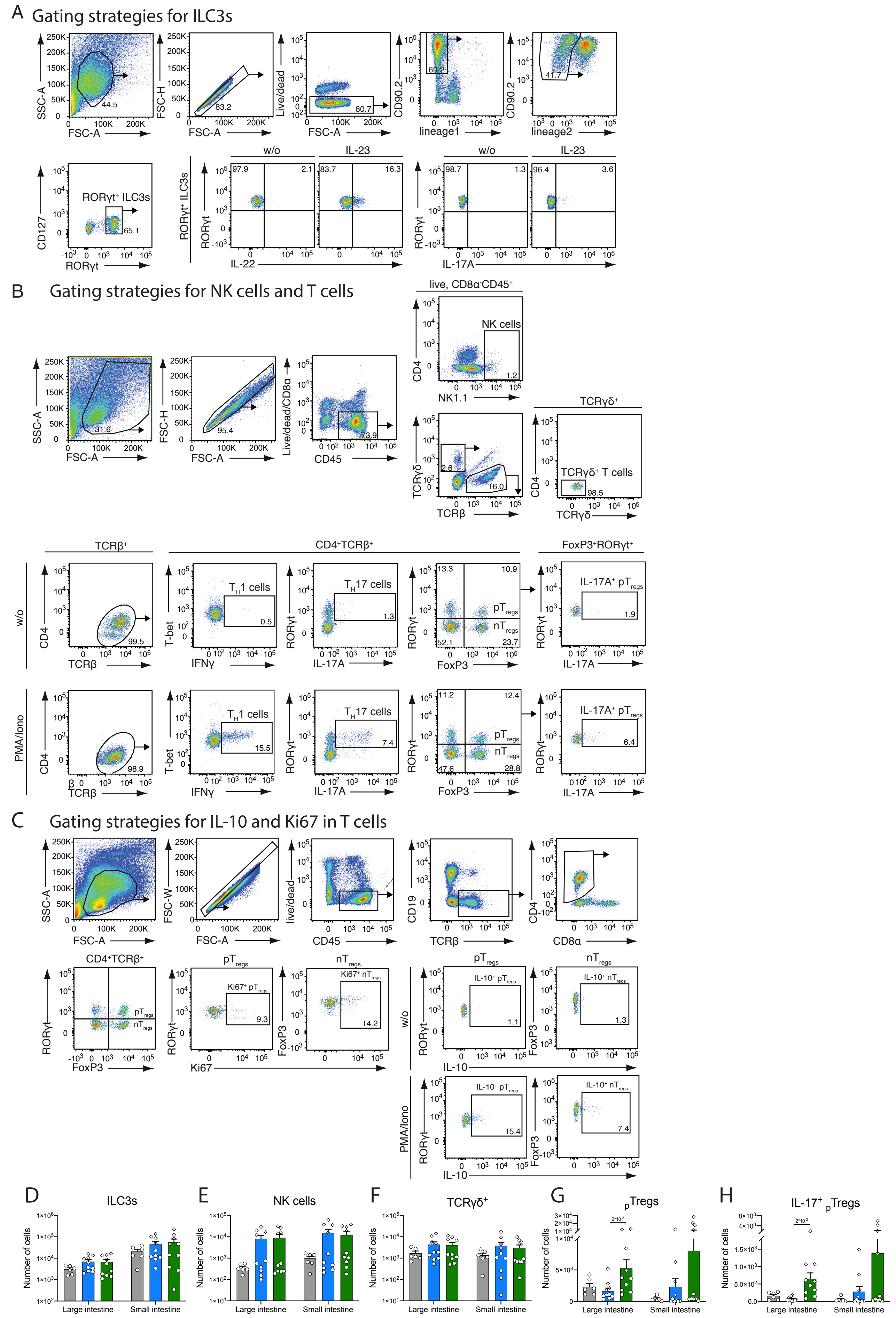

Supplementary Figure 3: Gating strategies and immune cell distribution in small and large intestine. A) Gating strategy for group 3 innate lymphoid cells (ILC3). Corresponds to Supplementary Figure 3D. Representative flow cytometry plots of *ex vivo* stimulated small intestine (SI) lamina propria (LP) cells. ILC3s were gated as single, live cells, lineage1$^-$lineage2$^-$ CD90.2$^+$CD127int/$^+$RORγt$^+$ cells. Representative data of RORγt+ ILC3s after stimulation with or without (w/o) IL-23 are shown. Lineage1: CD11c, CD19, CD45R/B220, Ly-6G/Ly-6C (Gr-1), Ter119, NK1.1; Lineage2: CD3ε, CD8α, TCRβ, TCR$\gamma\delta$. **B)** Gating strategy for NK cells and T cells. Corresponds to Supplementary Figure 3E-H; 4A-B; 5J. Representative flow cytometry plots of *ex vivo* stimulated colon lamina propria cells. NK cells were gated as single, live cells, CD8α^- CD45$^+$NK1.1$^+$; TCR$\gamma\delta^+$ T cells were gated as single, live cells, CD8α^-CD45$^+$TCR$\gamma\delta^+$CD4$^-$; CD4$^+$ T_H1 cells were gated as single, live cells, CD8α^-CD45$^+$TCRβ^+CD4$^+$T-bet$^+$IFNγ^+; CD4$^+$ T_H17 cells were gated as single, live cells, CD8α^-CD45$^+$TCRβ^+CD4$^+$RORγt$^+$IL-17A$^+$. Natural regulatory T cells ($_nT_{regs}$) were gated as single, live cells, CD8α^-CD45$^+$TCRβ^+CD4$^+$FoxP3$^+$RORγt$^-$ and peripheral-induced regulatory T cells ($_pT_{regs}$) were gated as single, live cells, CD8α^-CD45$^+$TCRβ^+CD4$^+$FoxP3$^+$RORγt$^+$. Representative data of TCRβ^+CD4$^+$ T cells after stimulation with or without (w/o) PMA and Ionomycin (PMA/Iono) are shown. **C)** Gating strategy for IL-10$^+$ and Ki67$^+$ $_{n}$- and $_p$Tregs. Corresponds to Figure 2O-R and Supplementary Figure 4C-F. Representative flow cytometry plots of *ex vivo* stimulated colon lamina propria cells. Natural and peripheral-induced regulatory T cells ($_nT_{regs}$ and $_pT_{regs}$, respectively) were gated as in (B), stained for Ki67 and IL-10; the latter with or without (w/o) PMA and Ionomycin (PMA/Iono). **D)** ILC3 cell number in large intestine (LI)-LP and SI-LP. **E)** NK1.1$^+$ NK cell number in LI-LP and SI-LP. **F)** TCR$\gamma\delta^+$ T cell number in LI-LP and SI-LP. **G)** Amount of $_pT_{regs}$ in LI-LP and SI-LP. **H)** Amount of IL17$^+$$_pT_{regs}$ in LI-LP and SI-LP. **D-H)** Bars represent mean ± SEM and dots individual data points. All p-values < 1*10^{-1} between WD_{CTNL} and indicated group are depicted. Dot shapes indicate individuals data points in Exp1 (squares) and Exp2 (circles). Grey bars: LFD-fed, n = 6; Blue bars: WD_{CNTL}-fed, n= 10; and Green bars: WD_{McB}-fed, n= 10. Statistical significant differences to WD_{CNTL} by one-way ANOVA, adjusted for multiple comparisons by Dunnett's post hoc.

Supplementary Figure 4

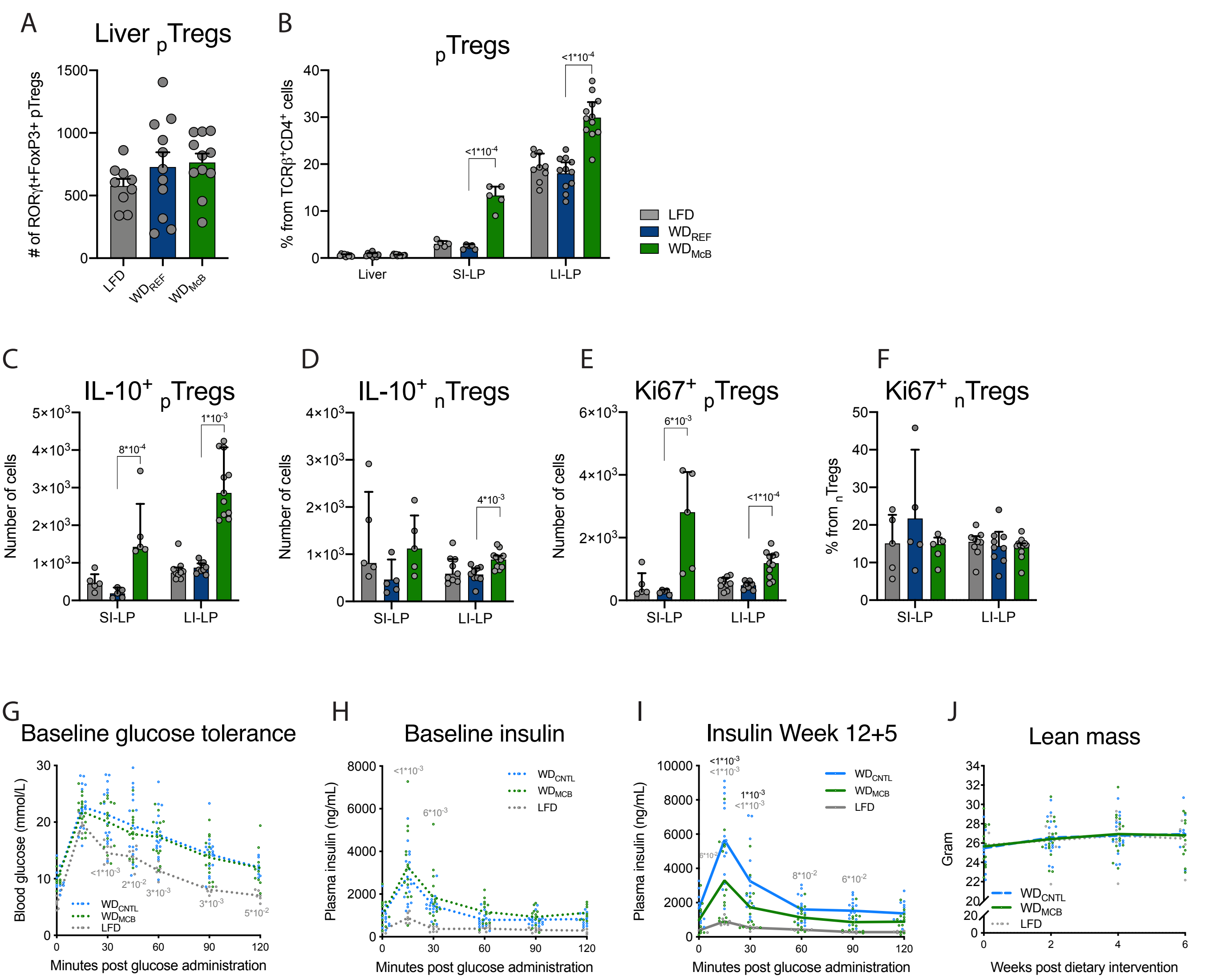

Supplementary Figure 4: Immunometabolic response to experimental diet feeding. A-B) Number of hepatic (A) $_{p}T_{regs}$ and their relative distribution between liver, SI-LP and LI-LP (B). Statistical significant differences to WD_{REF} by one-way ANOVA, adjusted for multiple comparisons by Dunnett's post hoc. In A, n = 9 (LFD) or 11 (WD_{REF} and WD_{McB}). In B, SI-LP n = 5 per group. Liver n = 9 (LFD and WD_{REF}) or 11 (WD_{McB}). LI-LP n = 9 (LFD), and 11 (WD_{REF}) or 12 (WD_{McB}). **C-F)** Amount of indicated cells in SI- and LI-LP from 'weight-matched' mice housed at thermoneutrality. Statistical significant differences to WD_{REF} by one-way ANOVA, adjusted for multiple comparisons by Dunnett's post hoc, except for LI-LP in C and F where statistical diffrences were evaluated by Kruskal-Wallis test, adjusted with Dunn's post hoc. SI-LP n = 5 per group. LI-LP n = 9 (LFD and WD_{REF}) or 11 (WD_{McB}). **G-H)** Blood glucose and plasma insulin values, respectively, during oral glucose tolerance test (OGTT) before dietary intervention (week 11). LFD n = 6, WD_{CNTL} and WD_{McB} n = 15 except in H at 60 min where WD_{CNTL} and WD_{McB} n = 14 as well as 90 and 120 min where WD_{McB} n = 12 due to insufficient sample material. **I)** Plasma insulin values from OGTT week 12+5. LFD n = 6, WD_{CNTL} and WD_{McB} n = 15. **J)** Lean mass in grams during the dietary intervention period as mean ± SEM of two independent experiments. LFD n = 6, WD_{CNTL} and WD_{McB} n = 15. **A-F)** Bars represent mean ± SEM and dots indicates individual data points. p-values $< 1*10^{-1}$ between WD_{REF} and indicated group are depicted. **G-J)** Lines represent mean and dots indicate individual data points from two independent experiments. p-values $< 1*10^{-1}$ between WD_{CNTL} and LFD (grey p-value), and WD_{CNTL} and WD_{McB} (black p-value) are depicted. Statistical significance compared to WD_{CNTL} by two-way ANOVA-RM, adjusted for multiple comparisons by Dunnett post hoc except for H where mixed effect analysis and Dunnetts post hoc test were used.

Supplementary Figure 5

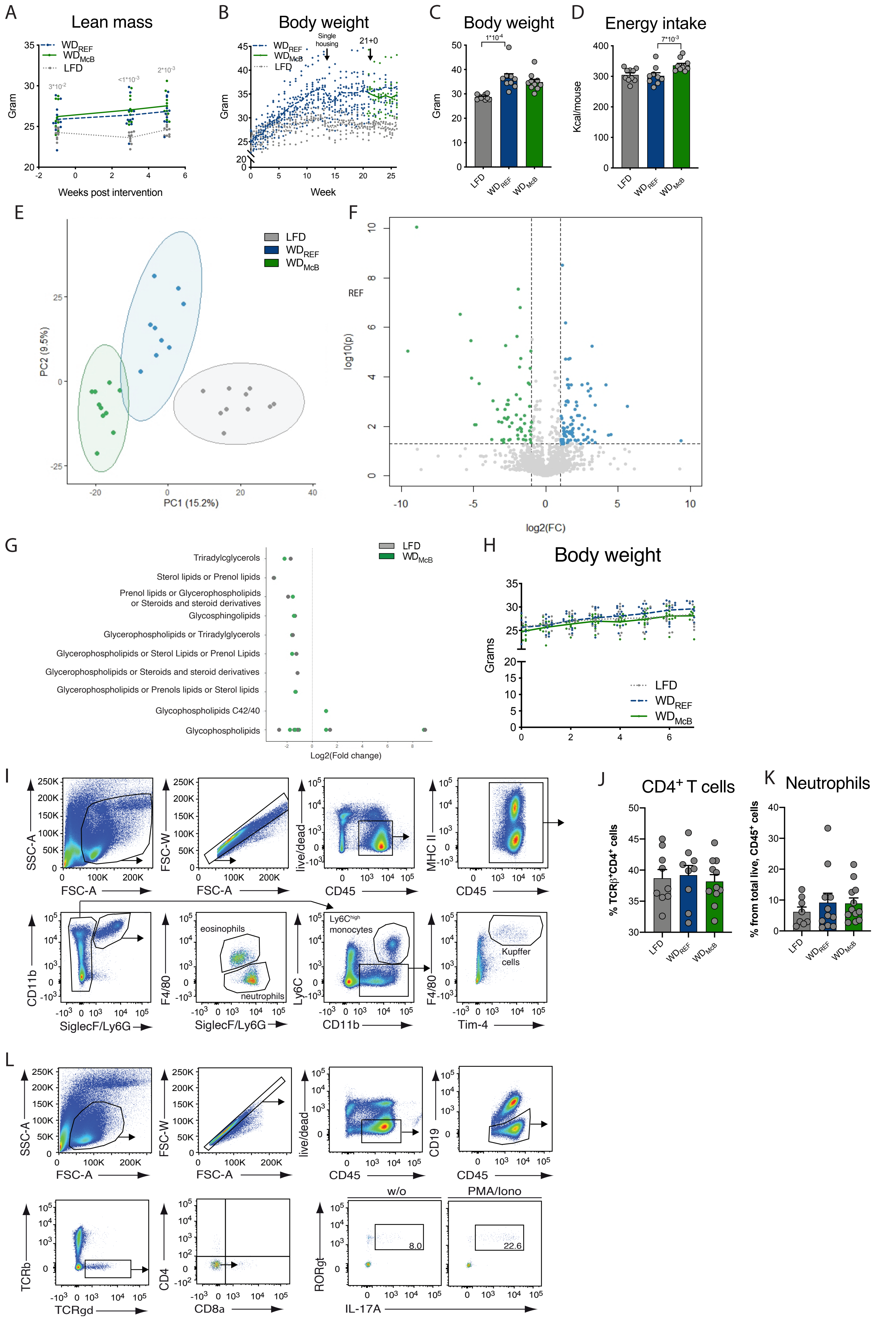

Supplementary Figure 5: Altered hepatic lipidome profile in response to WD_{McB} feeding. A) Lean mass in grams during the dietary intervention period. Lines represent mean and dots indicate individual data points. Statistical significance compared to WD_{REF} by two-way ANOVA-RM, adjusted for multiple comparisons by Dunnett post hoc. n = 10 per group. **B)** Body weight development; mice were fed either LFD or WD_{REF} for the first 21 weeks, as indicated. WD_{REF}-fed mice were subsequently stratified into experimental groups, fed indicated diets for additional 5 weeks. n = 10 per group. **C)** Final body weight week 21+5. Bars represents mean ± SEM and dots indicate individual data points. Statistical significant differences to WD_{REF} by one-way ANOVA, adjusted for multiple comparisons by Dunnett's post hoc. n = 10 (LFD and WD_{McB}) or 9 (WD_{REF}). **D)** Cumulative energy intake in kcal per mouse during dietary intervention (week 21+0 to 21+5). Bars represents mean ± SEM and dots indicate individual data points. Statistical significant differences to WD_{REF} by one-way ANOVA, adjusted for multiple comparisons by Dunnett's post hoc. n = 10 (LFD and WD_{McB}) or 9 (WD_{REF}). **E)** Principle coordinate analysis (PCoA) of hepatic lipid species identified in positive ionization mode. **F)** As in (E) but depicted in a volcano plot with significantly regulated lipid species (FDR < 0.05) presented on a log2 scale in either green ($WD_{McB} > WD_{REF}$) or blue ($WD_{McB} < WD_{REF}$). **G)** Lipid pathways identified by fold-change analysis seperating LFD (grey) and WD_{McB} (green) groups from WD_{REF} in positive ionization mode. **H)** Body weight development in 'weight-matched' WT mice fed the respective diets for 7 weeks. Lines represent mean and dots indicate individual datapoints. n = 9 (LFD), 11 (WD_{REF}) or 12 (WD_{McB}). **I)** Gating strategy of hepatic monocytes in mice from (H). Corresponds to Figure 5G, I-J; Supplementary Figure 5K. **J-K)** Amount of $CD4^{+}$ T cells and neutrophils in livers of 'weight-matched' WT mice fed the respective diets for 7 weeks. Bars represents mean ± SEM and dots indicate individual data points. Statistical significant differences to WD_{REF} by one-way ANOVA, adjusted for multiple comparisons by Dunnett's post hoc. n = 9 (LFD and WD_{REF}) or 11 (WD_{McB}). **L)** Gating strategy of hepatic IL-17+ γδ-T cells in mice from (H), gated as single live cells, $CD45^{+}CD19^{-}TCR\beta^{-}CD4^{-}$ $CD8\alpha^{-}TCR\gamma\delta^{+}ROR\gamma t^{+}IL\text{-}17A^{+}$. Corresponds to Figure 5K. Representative data of cells after stimulation with or without (w/o) PMA and Ionomycin (PMA/Iono) are shown.

Supplementary Figure 6

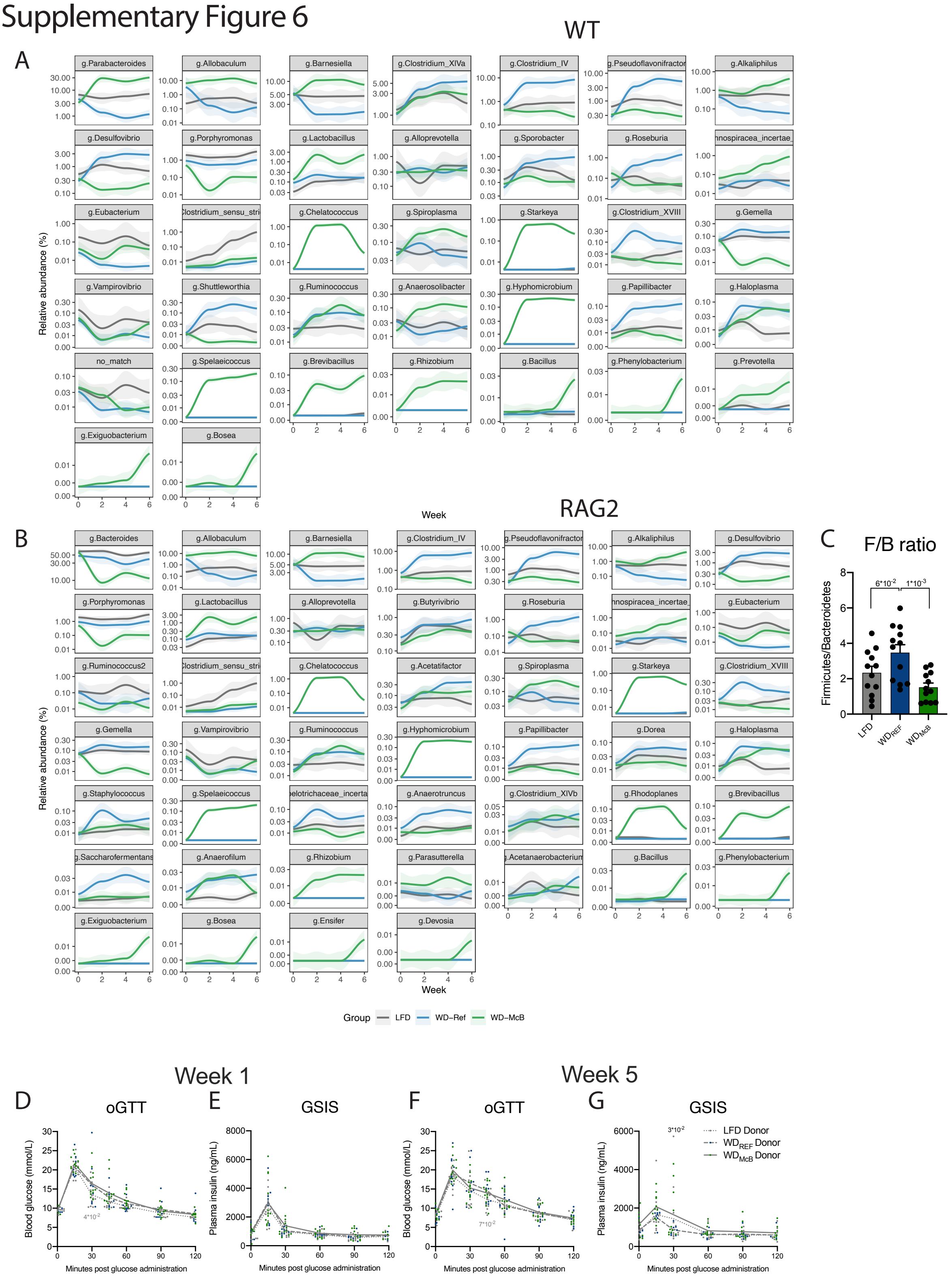

Supplementary Figure 6: Immunometabolism and gut microbiota interactions. A-B)) Deseq analysis of fecal bacterial genera abundances significantly regulated by McB intervention compared to the WD_{REF} (p.adj. < 0.05) in either WT (A) and RAG2$^{-/-}$ (B) mice. Relative abundance in % in each group and variation are shown for each regulated genus at the sampled time points. **C)** Firmicutes/Bacteroidetes ratio of fecal bacteria sampled after 6 weeks of experimental treatment of RAG2$^{-/-}$ mice. Bars represents mean ± SEM and dots indicate individual data points. Statistical significant differences to WD_{REF} by Kruskal-Wallis, adjusted for multiple comparisons by Dunn's post hoc. n = 12 per group. **D-G)** Blood glucose and plasma insulin values, respectively, during oral glucose tolerance test (OGTT) either 1 or 5 weeks after first cecal microbiota transfer in LFD-fed recipient mice. Lines represent mean and dots indicate individual datapoints. Statistical significant differences to WD_{REF} by mixed effect analysis and Dunnetts post hoc test. p-values < $1*10^{-1}$ between WD_{REF} and LFD (grey p-value), and WD_{REF} and WD_{McB} (black p-value) are depicted. **D-E)** n = 11 (LFD), 8 (WD_{REF}) and 12 (WD_{McB}), except for (E) time 0, where LFD = 8, WD_{REF} = 6, and WD_{McB} = 11, as well as time 60, 90 and 120 where WD_{McB} = 11. **F)** n = 12 (LFD and WD_{McB}) and 9 (WD_{REF}), except for time 120 where WD_{McB} = 11. **G)** n = 12 (LFD and WD_{McB}) and 7 (WD_{REF}), except for time 0-60 WD_{McB} = 11 and time 90-120 where LFD = 11 due to insufficient sample material.

Supplementary Table 1: Experimental diet compositions

		$WD_{No\ Protein}$	WD_{REF}	WD_{CNTL}	WD_{McB}	LFD
Corn starch	%	5,0000	5,0000	5,0000	5,0000	52,0000
Maltodextrin	%	10,0000	10,0000	10,0000	10,0000	--------
Sucrose	%	31,6300	31,6300	31,6300	31,6300	9,7270
Cellulose powder	%	5,0000	5,0000	5,0000	5,0000	5,0000
L-Cystine	%	0,3000	0,3000	0,3000	0,3000	0,3000
DL-Methionine	%	0,1600	0,1600	0,1600	0,1600	0,1600
Vitamin premix	%	1,0000	1,0000	1,0000	1,0000	1,0000
Mineral premix	%	6,0000	6,0000	6,0000	6,0000	3,5000
Choline chloride	%	0,2500	0,2500	0,2500	0,2500	0,2500
Vitamin K3	%	--------	--------	--------	--------	0,0005
Vitamin B12	%	--------	--------	--------	--------	0,0025
Butylated hydroxytoluene	%	0,0100	0,0100	0,0100	0,0100	0,0100
Cholesterol	%	0,1500	0,1500	0,1500	0,1500	--------
Na CMC	%	--------	--------	--------	--------	1,0000
Soybean oil	%	21,0000	21,0000	21,0000	21,0000	7,0000
Casein	%	--------	19,5000	16,5000	--------	20,0000
McB whole-cell lysate	%	--------	--------	--------	19,5000	--------
Macadamia oil	%	--------	--------	3,0000	--------	--------
Total	%	80,5	100,0	100,0	100,0	100,0

Supplementary Table 1: Composition of experimental diets used in the studies as % weight. Experimental diets were based on lot-matched WDNo Protein, and subsequently added casein, macadamia oil and McB whole-cell lysate as indicated.

Supplementary Table 2: Primer sequences and annealing temperature for qPCR

**Target gene**	**Forward primer sequence**	**Reverse primer sequence**	**Annealing temp. (°C)**
Scd1	ACA CCT GCC TCT TCG GGA TT	TGA TGC CCA GAG CGC TG	58
Acaca	TGC TGC CCC ATC CCC GGG	TCG AAC TCT CAC TGA CAC G	58
Acox1	GGG TCA TGG AAC	GAA TGA ACT CTT	58

	TCA TCT TCG A	GGG TCT TGG G	
Pparg1	GTG TGA CAG ACA AGA TTT GAA AG	GCT TGA TGT CAA AGG AAT GCG	60
Pparg2	ACA GCA AAT CTC TGT TTT ATG	TGC TGG AGA AAT CAA CTG TGG	60
Ppara	AGA GAG GAC AGA TGG GGC TC	CGT TTG TGG CTG GTC AAG TT	60

Supplementary Table 3: Deseq analysis from 12+6 week experiments

**Related to Figure 1**				
**Genus**	**Fold change**	**Log2FC**	**p value**	**Adj p value**
**g:Chelatococcus**	**1573,07**	**10,62**	**4,43E-25**	**2,88E-23**
**g:Barnesiella**	**27,46**	**4,78**	**3,07E-20**	**6,65E-19**
**g:Parabacteroides**	**23,89**	**4,58**	**2,22E-20**	**6,65E-19**
**g:Vampirovibrio**	**1401,99**	**10,45**	**3,08E-18**	**5,01E-17**
**g:Anaerofilum**	**408,29**	**8,67**	**6,55E-18**	**8,52E-17**
**g:Parasutterella**	**109,80**	**6,78**	**8,74E-16**	**9,47E-15**
**g:Microvirga**	**268,76**	**8,07**	**1,35E-14**	**1,25E-13**
**g:Brevibacillus**	**88,56**	**6,47**	**9,74E-09**	**7,36E-08**
**g:Pseudoxanthomonas**	**84,23**	**6,40**	**1,02E-08**	**7,36E-08**
**g:Clostridium_IV**	**0,24**	**-2,06**	**5,16E-08**	**3,35E-07**
**g:Isoptericola**	**51,65**	**5,69**	**5,17E-07**	**3,05E-06**
**g:Eubacterium**	**7,88**	**2,98**	**3,56E-06**	**1,93E-05**
**g:Akkermansia**	**632,32**	**9,30**	**7,69E-06**	**3,84E-05**
**g:Clostridium_XlVa**	**2,64**	**1,40**	**2,23E-05**	**0,0001**
**g:Staphylococcus**	**7,28**	**2,86**	**0,0002**	**0,001**
**g:Papillibacter**	**0,25**	**-2,01**	**0,0002**	**0,001**
**g:Acetatifactor**	**0,44**	**-1,19**	**0,001**	**0,002**
**g:Lachnospiracea_incertae_sedis**	**0,16**	**-2,61**	**0,001**	**0,002**

**g:Odoribacter**	**8,16**	**3,03**	**0,001**	**0,002**
**g:Bacteroides**	**2,81**	**1,49**	**0,002**	**0,01**
**g:Alistipes**	**2,83**	**1,50**	**0,003**	**0,01**
**g:Porphyromonas**	**4,77**	**2,25**	**0,004**	**0,01**
**g:Haloplasma**	**5,41**	**2,44**	**0,01**	**0,02**
**g:Desulfovibrio**	**0,44**	**-1,17**	**0,01**	**0,03**
**g:Blautia**	**0,24**	**-2,05**	**0,01**	**0,03**
**g:Escherichia/Shigella**	**0,14**	**-2,88**	**0,01**	**0,04**
**g:Murimonas**	**0,34**	**-1,57**	**0,02**	**0,04**
**g:Anaerovorax**	**3,76**	**1,91**	**0,02**	**0,04**
**g:Shuttleworthia**	**0,27**	**-1,87**	**0,02**	**0,05**
g:Alkaliphilus	0,15	-2,77	0,03	0,06
g:Dorea	0,41	-1,28	0,03	0,07
g:Allobaculum	2,17	1,12	0,04	0,07
g:Christensenella	8,11	3,02	0,04	0,07
g:Streptococcus	0,34	-1,57	0,03	0,07
g:Clostridium_XVIII	0,39	-1,36	0,04	0,07
g:Clostridium_III	2,16	1,11	0,04	0,07
g:Ruminococcus	0,42	-1,24	0,04	0,07
g:Pseudoflavonifractor	0,55	-0,86	0,05	0,08
g:Saccharofermentans	2,46	1,30	0,05	0,09
g:Lutispora	4,23	2,08	0,06	0,10
g:Butyrivibrio	1,75	0,81	0,08	0,12
g:Aestuariispira	3,27	1,71	0,09	0,15
g:Hydrogenoanaerobacterium	3,16	1,66	0,11	0,16
g:Anaerotruncus	0,55	-0,87	0,12	0,18
g:Enterorhabdus	0,46	-1,13	0,13	0,19
g:Flavonifractor	0,59	-0,76	0,14	0,19
g:Roseburia	0,54	-0,88	0,14	0,20
g:Sporobacter	1,95	0,96	0,15	0,21
g:Coprococcus	0,32	-1,62	0,17	0,22
g:Romboutsia	0,26	-1,95	0,26	0,34
g:Intestinimonas	0,59	-0,77	0,29	0,36
g:Butyricicoccus	0,34	-1,55	0,30	0,38
g:Marvinbryantia	1,69	0,75	0,35	0,44
g:Clostridium_XlVb	0,78	-0,35	0,38	0,46
g:Clostridium_sensu_stricto	0,51	-0,97	0,50	0,59
g:Gracilibacter	1,44	0,53	0,52	0,60
g:Catabacter	1,22	0,29	0,56	0,64
g:Anaerosporobacter	0,69	-0,53	0,60	0,67

g:Bilophila	0,79	-0,33	0,67	0,73
g:Enterococcus	1,46	0,54	0,67	0,73
g:Lactobacillus	1,17	0,23	0,73	0,78
g:Alloprevotella	1,17	0,23	0,75	0,79
g:Olsenella	1,13	0,17	0,84	0,86
g:Senegalimassilia	0,86	-0,21	0,85	0,86
g:Oscillibacter	1,01	0,01	0,99	0,99
**Related to Supplementary Figure 2**				
**Genus**	**Fold change**	**Log2FC**	**p value**	**Adj p value**
**g:Akkermansia**	**0,0007**	**-10,44**	**5,67E-35**	**4,08E-33**
**g:Bacillus**	**83,30**	**6,38**	**4,04E-15**	**1,45E-13**
**g:Acetatifactor**	**0,20**	**-2,30**	**9,26E-12**	**2,22E-10**
**g:Lactobacillus**	**32,59**	**5,03**	**1,43E-11**	**2,46E-10**
**g:Parasutterella**	**15,59**	**3,96**	**1,71E-11**	**2,46E-10**
**g:Clostridium_IV**	**0,17**	**-2,53**	**3,98E-11**	**4,78E-10**
**g:Nocardioides**	**52,24**	**5,71**	**9,07E-11**	**9,33E-10**
**g:Clostridium_XVIII**	**0,09**	**-3,54**	**3,02E-09**	**2,72E-08**
**g:Clostridium_XlVa**	**2,25**	**1,17**	**1,36E-08**	**1,09E-07**
**g:Sporobacter**	**0,21**	**-2,22**	**1,77E-08**	**1,28E-07**
**g:Brevibacillus**	**22,94**	**4,52**	**6,24E-08**	**4,08E-07**
**g:Eubacterium**	**4,77**	**2,25**	**9,72E-08**	**5,83E-07**
**g:Parabacteroides**	**4,21**	**2,07**	**4,94E-07**	**2,74E-06**
**g:Porphyromonas**	**3,77**	**1,92**	**5,48E-07**	**2,82E-06**
**g:Barnesiella**	**3,31**	**1,73**	**1,09E-06**	**5,25E-06**
**g:Enterococcus**	**26,51**	**4,73**	**1,43E-06**	**6,42E-06**
**g:Sphingopyxis**	**21,41**	**4,42**	**1,72E-06**	**7,28E-06**
**g:Anaerovorax**	**5,93**	**2,57**	**2,10E-05**	**8,41E-05**
**g:Haloplasma**	**3,38**	**1,76**	**9,88E-05**	**0,0004**
**g:Arthrobacter**	**9,18**	**3,20**	**0,0003**	**0,001**
**g:Rhizobium**	**8,90**	**3,15**	**0,0003**	**0,001**
**g:Vampirovibrio**	**6,25**	**2,64**	**0,0003**	**0,001**
**g:Allobaculum**	**4,86**	**2,28**	**0,0004**	**0,001**
**g:Neorhizobium**	**8,18**	**3,03**	**0,001**	**0,002**
**g:Paenibacillus**	**7,85**	**2,97**	**0,001**	**0,002**
**g:Romboutsia**	**4,91**	**2,29**	**0,001**	**0,002**
**g:Clostridium_sensu_stricto**	**12,44**	**3,64**	**0,001**	**0,002**
**g:Anaerotruncus**	**0,48**	**-1,07**	**0,001**	**0,004**
**g:Butyricicoccus**	**0,22**	**-2,21**	**0,001**	**0,004**
**g:Staphylococcus**	**6,33**	**2,66**	**0,002**	**0,01**

**g:Catenisphaera**	**7,26**	**2,86**	**0,002**	**0,01**
**g:Catabacter**	**0,47**	**-1,10**	**0,003**	**0,01**
**g:Papillibacter**	**0,43**	**-1,23**	**0,003**	**0,01**
**g:Coprobacillus**	**0,06**	**-4,00**	**0,01**	**0,01**
**g:Dorea**	**0,45**	**-1,16**	**0,01**	**0,02**
**g:Lutispora**	**2,96**	**1,56**	**0,02**	**0,04**
**g:Shuttleworthia**	**0,17**	**-2,54**	**0,02**	**0,04**
g:Alloprevotella	0,51	-0,98	0,03	0,05
g:Pseudoflavonifractor	0,63	-0,67	0,03	0,05
g:Hydrogenoanaerobacterium	2,49	1,32	0,03	0,06
g:Clostridium_XlVb	0,74	-0,44	0,08	0,14
g:Intestinimonas	1,79	0,84	0,08	0,14
g:Turicibacter	3,32	1,73	0,08	0,14
g:Prevotella	3,25	1,70	0,08	0,14
g:Erysipelotrichaceae_incertae_sedis	3,62	1,86	0,12	0,19
g:Roseburia	0,54	-0,88	0,14	0,21
g:Christensenella	2,02	1,01	0,18	0,27
g:Desulfovibrio	0,80	-0,33	0,23	0,34
g:Lachnospiracea_incertae_sedis	1,35	0,43	0,22	0,34
g:Murimonas	1,78	0,83	0,23	0,34
g:Anaerosporobacter	0,59	-0,76	0,25	0,35
g:Odoribacter	1,55	0,63	0,31	0,42
g:Enterorhabdus	1,47	0,56	0,33	0,45
g:Blautia	0,61	-0,71	0,37	0,47
g:Butyrivibrio	0,70	-0,51	0,37	0,47
g:Oscillibacter	1,23	0,30	0,37	0,47
g:Streptococcus	0,69	-0,54	0,36	0,47
g:Olsenella	0,53	-0,91	0,46	0,58
g:Bilophila	1,15	0,20	0,53	0,64
g:Coprococcus	1,32	0,40	0,54	0,64
g:Aestuariispira	0,73	-0,46	0,57	0,67
g:Bacteroides	0,84	-0,26	0,58	0,67
g:Ruminococcus2	2,00	1,00	0,58	0,67
g:Saccharofermentans	1,16	0,22	0,64	0,72
g:Gracilibacter	0,88	-0,18	0,75	0,83
g:Alkaliphilus	0,84	-0,25	0,77	0,84
g:Alistipes	0,94	-0,09	0,85	0,89
g:Flavonifractor	1,04	0,06	0,86	0,89
g:Gemella	0,90	-0,15	0,88	0,89
g:Ruminococcus	1,05	0,07	0,87	0,89

g:Senegalimassilia	0,90	-0,15	0,87	0,89
g:Clostridium_III	1,01	0,01	0,96	0,96

Results from deseq analysis of bacterial genera of the two experiments presented in Figure 1 & Supplementary Figure 2 featuring genus name, Fold change and Log2 fold change, comparing WD_{McB} to WD_{CNTL}. Unadjusted p-values as well as p-values adjusted by Benjamini-Hochberg method are reported and statistically significantly affected genera (Adj. $p < 0.05$) are marked in bold.

Supplementary Table 4: Lipid species altered by McB:

**Lipid**	**Adjusted p-value between WD_{REF} and LFD**	**Adjusted p-value between WD_{REF} and WD_{McB}**
**Upregulated by McB:**		
lysoPC 17:1	0,7072	0,0022
PC 33:1 B	0,5237	<0,0001
PC 33:5 D	0,0001	<0,0001
PC 38:4 C	<0,0001	<0,0001
PC 39:6	0,0021	<0,0001
PE 38:5	0,0004	0,0081
plasmenyl-PC 36:3	0,0002	0,0004
**Downregulated by McB:**		
lysoPC 16:1	0,9977	0,0023
lysoPC 18:1	0,4575	0,0065
lysoPC 20:1	0,0003	0,0001
lysoPC 20:2	0,0027	0,0028
lysoPC 20:3	0,0019	0,0005
lysoPC 20:4 A	0,9830	0,0018
PC 32:1	0,0004	0,0012
PC 33:5 C	0,0933	<0,0001
PC 34:3	0,4656	0,0001
PC 35:4	<0,0001	0,0003
PC 36:3 A	0,0003	0,0002
PC 36:3 B	<0,0001	<0,0001
PC 36:5 B	0,9151	<0,0001
PC 37:4 A	0,0009	0,0085
PC 38:4 A	0,0001	0,0004
PC 38:4 B	<0,0001	<0,0001
PC 38:5 A	0,2436	<0,0001
PC 38:6 B	0,0006	<0,0001
PC 39:7	0,0022	0,0033
PC 40:7 B	0,0006	0,0001

Reported p-values compared to the WD_{REF} are obtained by mixed-effects analysis, adjusted for multiple comparisons by Dunnett's post hoc.

Supplementary Table 5: Deseq analysis from 21+5 week experiment

Related to Figure 6				
**Genus**	**Fold change**	**Log2FC**	**p value**	**Adj p value**
**g:Parasutterella**	**2066,66**	**11,013**	**1,4E-38**	**9,3E-37**
**g:Parabacteroides**	**94,91**	**6,568**	**7,6E-33**	**2,6E-31**
**g:Brevibacillus**	**138,42**	**7,113**	**9,1E-18**	**2,1E-16**
**g:Pseudoflavonifractor**	**0,09**	**-3,418**	**3,4E-14**	**5,9E-13**
**g:Odoribacter**	**19,74**	**4,303**	**1,9E-13**	**2,6E-12**
**g:Desulfovibrio**	**0,18**	**-2,501**	**4,8E-09**	**5,5E-08**
**g:Haloplasma**	**13,78**	**3,785**	**7,0E-09**	**6,9E-08**
**g:Dorea**	**0,11**	**-3,168**	**1,3E-08**	**9,9E-08**
**g:Flavonifractor**	**0,11**	**-3,153**	**1,3E-08**	**9,9E-08**
**g:Acetatifactor**	**0,16**	**-2,621**	**2,3E-07**	**1,6E-06**
**g:Rhodococcus**	**40,07**	**5,325**	**3,5E-07**	**2,2E-06**
**g:Romboutsia**	**0,04**	**-4,556**	**2,9E-06**	**1,7E-05**
**g:Alistipes**	**3,95**	**1,982**	**4,3E-06**	**2,3E-05**
**g:Porphyromonas**	**8,17**	**3,031**	**1,5E-05**	**7,4E-05**
**g:Shuttleworthia**	**0,09**	**-3,499**	**1,6E-05**	**7,4E-05**
**g:Allobaculum**	**2,74**	**1,454**	**1,0E-04**	**4,3E-04**
**g:Barnesiella**	**2,88**	**1,524**	**1,1E-04**	**4,7E-04**
**g:Anaerotruncus**	**0,24**	**-2,039**	**2,0E-04**	**7,5E-04**
**g:Akkermansia**	**8,07**	**3,013**	**3,5E-04**	**1,3E-03**
**g:Mucispirillum**	**0,14**	**-2,789**	**4,8E-04**	**1,7E-03**
**g:Cohnella**	**15,91**	**3,992**	**9,6E-04**	**3,1E-03**
**g:Clostridium_IV**	**0,51**	**-0,966**	**1,2E-03**	**3,6E-03**
**g:Clostridium_XlVa**	**0,54**	**-0,893**	**1,3E-03**	**3,8E-03**
**g:Eubacterium**	**4,77**	**2,253**	**1,6E-03**	**4,5E-03**
**g:Papillibacter**	**0,27**	**-1,874**	**2,1E-03**	**5,8E-03**
**g:Coprobacter**	**13,09**	**3,710**	**3,0E-03**	**7,8E-03**
**g:Oscillibacter**	**0,38**	**-1,415**	**3,2E-03**	**8,2E-03**
**g:Bifidobacterium**	**7,60**	**2,926**	**4,4E-03**	**1,1E-02**
**g:Sporobacter**	**0,39**	**-1,364**	**1,0E-02**	**2,5E-02**
**g:Clostridium_XVIII**	**0,36**	**-1,481**	**1,2E-02**	**2,8E-02**
**g:Ruminococcus**	**0,44**	**-1,178**	**1,6E-02**	**3,6E-02**

**g:Christensenella**	**4,25**	**2,086**	**2,3E-02**	**4,9E-02**
g:Clostridium_XlVb	2,63	1,395	0,025	0,050
g:Hydrogenoanaerobacterium	0,25	-2,005	0,029	0,058
g:Streptococcus	0,38	-1,406	0,033	0,064
g:Butyrivibrio	1,64	0,715	0,041	0,077
g:Lactobacillus	2,02	1,018	0,046	0,083
g:Olsenella	2,39	1,260	0,069	0,123
g:Butyricicoccus	0,31	-1,678	0,079	0,137
g:Blautia	0,47	-1,096	0,113	0,182
g:Pedobacter	3,78	1,918	0,112	0,182
g:Roseburia	0,52	-0,954	0,111	0,182
g:Murimonas	0,28	-1,855	0,129	0,203
g:Fodinicurvata	3,54	1,822	0,140	0,214
g:Holdemania	1,96	0,970	0,150	0,225
g:Clostridium_III	1,42	0,506	0,184	0,264
g:Intestinimonas	0,54	-0,877	0,183	0,264
g:Anaerofilum	0,44	-1,168	0,191	0,269
g:Anaerosolibacter	0,32	-1,630	0,208	0,287
g:Coprococcus	2,06	1,042	0,242	0,327
g:Erysipelotrichaceae_incertae_sedis	0,44	-1,194	0,323	0,428
g:Bilophila	0,68	-0,546	0,329	0,428
g:Anaerovorax	1,79	0,843	0,352	0,449
g:Enterococcus	2,38	1,251	0,366	0,459
g:Staphylococcus	0,61	-0,707	0,379	0,468
g:Escherichia/Shigella	0,42	-1,250	0,411	0,498
g:Bacteroides	0,82	-0,293	0,420	0,500
g:Lachnospiracea_incertae_sedis	0,75	-0,408	0,469	0,548
g:Lutispora	1,83	0,871	0,493	0,567
g:Alkaliphilus	1,55	0,632	0,521	0,589
g:Clostridium_sensu_stricto	1,60	0,680	0,562	0,625
g:Enterorhabdus	1,27	0,346	0,573	0,625
no_match	2,30	1,202	0,579	0,625
g:Gracilibacter	1,23	0,304	0,591	0,627
g:Saccharofermentans	1,12	0,167	0,773	0,808
g:Anaerosporobacter	0,97	-0,047	0,938	0,952
g:Tissierella	1,06	0,078	0,938	0,952
g:Spiroplasma	1,03	0,038	0,969	0,969

Results from deseq analysis of bacterial genera of the 21+5 week experiment featuring genus name, Log2 fold change, comparing WD_{McB} to WD_{REF}. Unadjusted p-values as well as p-values adjusted by Benjamini-Hochberg method are reported and statistically significantly affected genera (Adj. $p < 0.05$) are marked in bold.
