# Supplementary material for: Lysates of Methylococcus capsulatus Bath induce a lean-like microbiota, intestinal FoxP3+RORγt+IL-17+ Tregs and improve metabolism
Source: Nat Commun. 2021 Feb 17;12:1093. doi: 10.1038/s41467-021-21408-9 (PMC7889900; doi:10.1038/s41467-021-21408-9)
Supplement: Supplementary file 3 — Reporting summary [file 41467_2021_21408_MOESM3_ESM.pdf]

nature research

Corresponding author(s): Benjamin A. H. Jensen
Karsten Kristiansen
Tor. E. Lea

Last updated by author(s): Jan 8, 2021

Reporting Summary

Nature Research wishes to improve the reproducibility of the work that we publish. This form provides structure for consistency and transparency in reporting. For further information on Nature Research policies, see our Editorial Policies and the Editorial Policy Checklist.

Statistics

For all statistical analyses, confirm that the following items are present in the figure legend, table legend, main text, or Methods section.

n/a	Confirmed	
☐	☒	The exact sample size (n) for each experimental group/condition, given as a discrete number and unit of measurement
☐	☒	A statement on whether measurements were taken from distinct samples or whether the same sample was measured repeatedly
☐	☒	The statistical test(s) used AND whether they are one- or two-sided<br>*Only common tests should be described solely by name; describe more complex techniques in the Methods section.*
☐	☒	A description of all covariates tested
☐	☒	A description of any assumptions or corrections, such as tests of normality and adjustment for multiple comparisons
☐	☒	A full description of the statistical parameters including central tendency (e.g. means) or other basic estimates (e.g. regression coefficient) AND variation (e.g. standard deviation) or associated estimates of uncertainty (e.g. confidence intervals)
☐	☒	For null hypothesis testing, the test statistic (e.g. F, t, r) with confidence intervals, effect sizes, degrees of freedom and P value noted<br>*Give P values as exact values whenever suitable.*
☒	☐	For Bayesian analysis, information on the choice of priors and Markov chain Monte Carlo settings
☒	☐	For hierarchical and complex designs, identification of the appropriate level for tests and full reporting of outcomes
☒	☐	Estimates of effect sizes (e.g. Cohen's d, Pearson's r), indicating how they were calculated

Our web collection on statistics for biologists contains articles on many of the points above.

Software and code

Policy information about availability of computer code

Data collection	We did not collect 'external' data for this manuscript, and so no software were used for such collection.
Data analysis	To select OTUs and species enriched in different subgroups, DESeq2v1.29 with default settings was used in the differential abundance testing. Genera with p.adj. < 0.05 comparing the last sampling point for WDREF/WDCNTL vs WDMcB with DESeq2v1.29 differential expression analysis based on the negative binomial distribution were reported. Permutational multivariate analysis of variance (permanova) using weighted UniFrac distance matrices (Adonis, Vegan v2.5 R v4.0 package) was used to evaluate overall microbial compositions.<br>Normality residuals of remaining data were assessed by D'Augostino-Pearson omnibus (k2), Graphpad Prism 8, and subsequently evaluated by parametric (gaussian distributed) or non-parametric (non-gaussian distributed) tests as appropriate. Details of each test including post hoc assessment are specified in figure legends. Data are expressed as mean ± SEM with individual dots, and all groups are compared to the relevant WDREF or WDCNTL group as indicated. .

For manuscripts utilizing custom algorithms or software that are central to the research but not yet described in published literature, software must be made available to editors and reviewers. We strongly encourage code deposition in a community repository (e.g. GitHub). See the Nature Research guidelines for submitting code & software for further information.

Data

Policy information about availability of data

All manuscripts must include a data availability statement. This statement should provide the following information, where applicable:

- Accession codes, unique identifiers, or web links for publicly available datasets
- A list of figures that have associated raw data
- A description of any restrictions on data availability

Data availability:

April 2020

16S sequencing data is uploaded to a public repository with study accession number: PRJEB41983 and available at the following link: http://www.ebi.ac.uk/ena/data/view/PRJEB41983. All non-omics datasets included in the current study are available in the figshare repository Jensen, B. A. H. et al. Data underlying the manuscript: Lysates of Methylococcus capsulatus Bath induce a lean-like microbiota, intestinal FoxP3+RORγt+IL-17+ Tregs and improve metabolism. figshare https://doi.org/10.6084/m9.figshare.13522283 (2021).
Code availability:
Standard codes from the cited R-packages were used in this manuscript. Specific scripts are available upon reasonable request

Field-specific reporting

Please select the one below that is the best fit for your research. If you are not sure, read the appropriate sections before making your selection.

- [x] Life sciences
- [ ] Behavioural & social sciences
- [ ] Ecological, evolutionary & environmental sciences

For a reference copy of the document with all sections, see nature.com/documents/nr-reporting-summary-flat.pdf

Life sciences study design

All studies must disclose on these points even when the disclosure is negative.

Sample size

We did not perform specific power calculations in this study, but instead used a sufficiently large n-size as per experience. CMT studies were limited by available amount of cecal content to transfer. The n-size was thus restricted accordingly.

Data exclusions

Three WT mice (two WD-REF-fed and 1 WD-MvcB-fed) were excluded for immunological assessment by flow cytometry in the third experiment on hepatic and gastrointestinal immune profiling (Figure 2O-R, 5I-K, S4C-F, S5J-K), due to visual wounds on their legs and/or back. These wounds were a result of hierarchical dominance within the cages and only evident for the last few days of the experiment for which reason, they were included in all other analysis. One LFD-fed mouse were excluded from the hepatic immune assessment (also by flow cytometry), due to mishandling of the single cell suspension.
The following samples were excluded and/or missing from insulin analyzes during oral glucose tolerance test: one WD-REF fed RAG2 mouse were excluded at T15 by pre-established outlier criteria (~10-fold increased compared to all other mice at T15) and one WD-McB fed mouse were excluded at T60 due to hemolysis (pre-established exclusion criteria). Lastly, several T0 samples from the CMT experiments were below LOD, just as some time points were missing sample material, as indicted in the figure legends and also evident from the raw data deposited at figshare (link in the data availability section).

Replication

As specified throughout the manuscript, the general findings reported here has been replicated in several mouse strains, at different experimental duration and at different temperatures. All replication attempts were successfully achieved. Data supporting the main conclusion has been replicated at least twice. All data from such replication studies are explicitly shown and discussed. When applicable, data from replication studies are represented in the same bar plots, where the shape of each dote are experiment-dependent. This was deliberately done to corroborate and visualize the extraordinary level of reproducibility in the reported phenotype.

Randomization

For all therapeutic intervention studies, mice were stratified into treatment groups based on their 'baseline' body weight and glucoregulatory capacity, as described in the manuscript.
For all prophylactic experiments, mice were randomly assigned to experimental groups.

Blinding

All mice were assigned a random number as mouse ID. Staff handling the mice and/or performing specific analyzes only knew this ID and were thus blinded for group ID during experimental handling (e.g. OGTT and GSIS) and data processing (e.g. SCFAs, FLow cytometry etc.).

Reporting for specific materials, systems and methods

We require information from authors about some types of materials, experimental systems and methods used in many studies. Here, indicate whether each material, system or method listed is relevant to your study. If you are not sure if a list item applies to your research, read the appropriate section before selecting a response.

Materials & experimental systems

n/a	Involved in the study	
☐	☒	Antibodies
☒	☐	Eukaryotic cell lines
☒	☐	Palaeontology and archaeology
☐	☒	Animals and other organisms
☒	☐	Human research participants
☒	☐	Clinical data
☒	☐	Dual use research of concern

Methods

n/a	Involved in the study	
☒	☐	ChIP-seq
☐	☒	Flow cytometry
☒	☐	MRI-based neuroimaging

Antibodies

Antibodies used

anti-CD3-Biotin (clone: 17A2) BioLegend cat#: 100244 (dilution: 1/250)
anti-CD4-Pacific Blue (clone: GK1.5) BioLegend cat#: 100427 (dilution: 1/200)
anti-CD4-APC-eFluor780 (clone: GK1.5) eBioscience cat#: 47-0042-82 (dilution: 1/200)
anti-CD4-BUV395 (clone: GK1.5) BD Biosciences cat#: 563790 (dilution: 1/200)
anti-CD8-BV510 (clone: 53-6.7) BioLegend cat#: 100751 (dilution: 1/200)
anti-CD8-Biotin (clone: 53-6.7) eBioscience cat#: 13-0081-81 (dilution: 1/250)
anti-CD8-BV480 (clone: 53-6.7) BD Biosciences cat#: 566096 (dilution: 1/200)
anti-CD8-BV785/786 (clone: 53-6.7) BD Biosciences cat#: 563332 (dilution: 1/200)
anti-CD11b-AlexaFluor 647 (clone: M1/70) BioLegend cat#: 101220 (dilution: 1/300)
anti-CD11c-AlexaFluor 700 (clone: N418) BioLegend cat#: 117320 (dilution: 1/200)
anti-CD19-AlexaFluor 700 (clone: 6D5) BioLegend cat#: 115528 (dilution: 1/100)
anti-CD19-BV711 (clone: 1D3) BD Biosciences cat#: 563157 (dilution: 1/200)
anti-CD45-AlexaFluor 700 (clone: 30-F11) eBioscience cat#: 56-0451-80 (dilution: 1/200)
anti-CD45-APCCy7 (clone: 30-F11) BD Biosciences cat#: 557659 (dilution: 1/200)
anti-CD45R/B220-AlexaFluor 700 (clone: RA3-6B2) BioLegend cat#: 103232 (dilution: 1/200)
anti-CD90.2-PECy7 (clone: 30-H12) BioLegend cat#: 105325 (dilution: 1/200)
anti-CD127-BV605 (clone: A7R34) BioLegend cat#: 135025 (dilution: 1/60)
anti-Ly-6G/Ly-6C (Gr-1)-AlexaFluor 700 (RB6-8C5) BioLegend cat#: 108422 (dilution: 1/200)
anti-Ly-6C-PECy7 (clone: AL-21) BD Biosciences cat#: 560593 (dilution: 1/200)
anti-Ly-6G-AlexaFluor 700 (clone: 1A8) BD Biosciences cat#: 561236 (dilution: 1/200)
anti-TER119-AlexaFluor 700 (clone: TER-119) BioLegend cat#: 116220 (dilution: 1/200)
anti-F4/80-Biotin (clone: BM8) eBioscience cat#: 13-4801-81 (dilution: 1/600)
anti-I-A/I-E-BV605 (clone: M5/114.15.2) BD Biosciences cat#: 563413 (dilution: 1/600)
anti-Siglec-F-APC-R700 (clone: E50-2440) BD Biosciences cat#: 565183 (dilution: 1/200)
anti-TIM-4-PE (clone: 54(RMT4-54)) eBioscience cat#: 12-5866-80 (dilution: 1/200)
anti-TCR-AlexaFluor (clone: H57-597) BioLegend cat#: 109224 (dilution: 1/100)
anti-TCR-PECF594 (clone: H57-597) BD Biosciences cat#: 562841 (dilution: 1/200)
anti-TCR-Biotin (clone: GL3) BioLegend cat#: 118103 (dilution: 1/250)
anti-TCR-BV421 (clone: GL3) BioLegend cat#: 118119 (dilution: 1/200)
anti-NK1.1-AlexaFluor 700 (clone: PK136) BioLegend cat#: 108730 (dilution: 1/200)
anti-NK1.1-BV785 (clone: PK136) BioLegend cat#: 108749 (dilution: 1/200)
anti-NKp46-BV786 (clone: 29A1.4) BD Biosciences cat#: 741029 (dilution: 1/200)
anti-FoxP3-APC (clone: FJK-16s) eBioscience cat#: 17-5773-80 (dilution: 1/200)
anti-FoxP3-PEeFluor610 (clone: FJK-16s) eBioscience cat#: 61-5773-82 (dilution: 1/200)
anti-RORt-PE (clone: B2D) eBioscience cat#: 12-6981-80 (dilution: 1/200)
anti-RORt-PE-eFluor610 (clone: B2D) eBioscience cat#: 61-6981-80 (dilution: 1/200)
anti-T-bet-PECy7 (clone: 4B10) eBioscience cat#: 25-5825-81 (dilution: 1/200)
anti-Ki67-BV786 (clone: B56) BD Biosciences cat#: 563756 (dilution: 1/100)
anti-IL-10-PE (clone: JES5-16E3) eBioscience cat#: 12-7101-82 (dilution: 1/200)
anti-IL-17A-AlexaFluor 488 (clone: TC11-18H10.1) BioLegend cat#: 506909 (dilution: 1/200)
anti-IL-22-PerCP-eFluor710 (clone: IL22JOP) eBioscience cat#: 46-7222-80 (dilution: 1/100)
anti-IFN-BV605 (clone: XMG1.2) BioLegend cat#: 505839 (dilution: 1/200)
Streptavidin-PE-CF594 BD Biosciences cat#: 562284 (dilution: 1/500)
Streptavidin-BV421 BioLegend cat#: 405226 (dilution: 1/250)
anti-CD3-Biotin (clone: 17A2) BioLegend cat#: 100244 (dilution: 1/250)
anti-CD4-Pacific Blue (clone: GK1.5) BioLegend cat#: 100427 (dilution: 1/200)
anti-CD4-APC-eFluor780 (clone: GK1.5) eBioscience cat#: 47-0042-82 (dilution: 1/200)
anti-CD4-BUV395 (clone: GK1.5) BD Biosciences cat#: 563790 (dilution: 1/200)
anti-CD8-BV510 (clone: 53-6.7) BioLegend cat#: 100751 (dilution: 1/200)
anti-CD8-Biotin (clone: 53-6.7) eBioscience cat#: 13-0081-81 (dilution: 1/250)
anti-CD8-BV480 (clone: 53-6.7) BD Biosciences cat#: 566096 (dilution: 1/200)
anti-CD8-BV785/786 (clone: 53-6.7) BD Biosciences cat#: 563332 (dilution: 1/200)
anti-CD11b-AlexaFluor 647 (clone: M1/70) BioLegend cat#: 101220 (dilution: 1/300)
anti-CD11c-AlexaFluor 700 (clone: N418) BioLegend cat#: 117320 (dilution: 1/200)
anti-CD19-AlexaFluor 700 (clone: 6D5) BioLegend cat#: 115528 (dilution: 1/100)
anti-CD19-BV711 (clone: 1D3) BD Biosciences cat#: 563157 (dilution: 1/200)
anti-CD45-AlexaFluor 700 (clone: 30-F11) eBioscience cat#: 56-0451-80 (dilution: 1/200)
anti-CD45-APCCy7 (clone: 30-F11) BD Biosciences cat#: 557659 (dilution: 1/200)
anti-CD45R/B220-AlexaFluor 700 (clone: RA3-6B2) BioLegend cat#: 103232 (dilution: 1/200)
anti-CD90.2-PECy7 (clone: 30-H12) BioLegend cat#: 105325 (dilution: 1/200)
anti-CD127-BV605 (clone: A7R34) BioLegend cat#: 135025 (dilution: 1/60)
anti-Ly-6G/Ly-6C (Gr-1)-AlexaFluor 700 (RB6-8C5) BioLegend cat#: 108422 (dilution: 1/200)
anti-Ly-6C-PECy7 (clone: AL-21) BD Biosciences cat#: 560593 (dilution: 1/200)
anti-Ly-6G-AlexaFluor 700 (clone: 1A8) BD Biosciences cat#: 561236 (dilution: 1/200)
anti-TER119-AlexaFluor 700 (clone: TER-119) BioLegend cat#: 116220 (dilution: 1/200)

anti-F4/80-Biotin (clone: BM8) eBioscience cat#: 13-4801-81 (dilution: 1/600)
anti-I-A/I-E-BV605 (clone: M5/114.15.2) BD Biosciences cat#: 563413 (dilution: 1/600)
anti-Siglec-F-APC-R700 (clone: E50-2440) BD Biosciences cat#: 565183 (dilution: 1/200)
anti-TIM-4-PE (clone: 54(RMT4-54)) eBioscience cat#: 12-5866-80 (dilution: 1/200)
anti-TCR-AlexaFluor (clone: H57-597) BioLegend cat#: 109224 (dilution: 1/100)
anti-TCR-PECF594 (clone: H57-597) BD Biosciences cat#: 562841 (dilution: 1/200)
anti-TCR-Biotin (clone: GL3) BioLegend cat#: 118103 (dilution: 1/250)
anti-TCR-BV421 (clone: GL3) BioLegend cat#: 118119 (dilution: 1/200)
anti-NK1.1-AlexaFluor 700 (clone: PK136) BioLegend cat#: 108730 (dilution: 1/200)
anti-NK1.1-BV785 (clone: PK136) BioLegend cat#: 108749 (dilution: 1/200)
anti-NKp46-BV786 (clone: 29A1.4) BD Biosciences cat#: 741029 (dilution: 1/200)
anti-FoxP3-APC (clone: FJK-16s) eBioscience cat#: 17-5773-80 (dilution: 1/200)
anti-FoxP3-PEeFluor610 (clone: FJK-16s) eBioscience cat#: 61-5773-82 (dilution: 1/200)
anti-RORt-PE (clone: B2D) eBioscience cat#: 12-6981-80 (dilution: 1/200)
anti-RORt-PE-eFluor610 (clone: B2D) eBioscience cat#: 61-6981-80 (dilution: 1/200)
anti-T-bet-PECy7 (clone: 4B10) eBioscience cat#: 25-5825-81 (dilution: 1/200)
anti-Ki67-BV786 (clone: B56) BD Biosciences cat#: 563756 (dilution: 1/100)
anti-IL-10-PE (clone: JES5-16E3) eBioscience cat#: 12-7101-82 (dilution: 1/200)
anti-IL-17A-AlexaFluor 488 (clone: TC11-18H10.1) BioLegend cat#: 506909 (dilution: 1/200)
anti-IL-22-PerCP-eFluor710 (clone: IL22JOP) eBioscience cat#: 46-7222-80 (dilution: 1/100)
anti-IFN-BV605 (clone: XMG1.2) BioLegend cat#: 505839 (dilution: 1/200)
Streptavidin-PE-CF594 BD Biosciences cat#: 562284 (dilution: 1/500)
Streptavidin-BV421 BioLegend cat#: 405226 (dilution: 1/250)
anti-CD3-Biotin (clone: 17A2) BioLegend cat#: 100244 (dilution: 1/250)
anti-CD4-Pacific Blue (clone: GK1.5) BioLegend cat#: 100427 (dilution: 1/200)
anti-CD4-APC-eFluor780 (clone: GK1.5) eBioscience cat#: 47-0042-82 (dilution: 1/200)
anti-CD4-BUV395 (clone: GK1.5) BD Biosciences cat#: 563790 (dilution: 1/200)
anti-CD8-BV510 (clone: 53-6.7) BioLegend cat#: 100751 (dilution: 1/200)
anti-CD8-Biotin (clone: 53-6.7) eBioscience cat#: 13-0081-81 (dilution: 1/250)
anti-CD8-BV480 (clone: 53-6.7) BD Biosciences cat#: 566096 (dilution: 1/200)
anti-CD8-BV785/786 (clone: 53-6.7) BD Biosciences cat#: 563332 (dilution: 1/200)
anti-CD11b-AlexaFluor 647 (clone: M1/70) BioLegend cat#: 101220 (dilution: 1/300)
anti-CD11c-AlexaFluor 700 (clone: N418) BioLegend cat#: 117320 (dilution: 1/200)
anti-CD19-AlexaFluor 700 (clone: 6D5) BioLegend cat#: 115528 (dilution: 1/100)
anti-CD19-BV711 (clone: 1D3) BD Biosciences cat#: 563157 (dilution: 1/200)
anti-CD45-AlexaFluor 700 (clone: 30-F11) eBioscience cat#: 56-0451-80 (dilution: 1/200)
anti-CD45-APCCy7 (clone: 30-F11) BD Biosciences cat#: 557659 (dilution: 1/200)
anti-CD45R/B220-AlexaFluor 700 (clone: RA3-6B2) BioLegend cat#: 103232 (dilution: 1/200)
anti-CD90.2-PECy7 (clone: 30-H12) BioLegend cat#: 105325 (dilution: 1/200)
anti-CD127-BV605 (clone: A7R34) BioLegend cat#: 135025 (dilution: 1/60)
anti-Ly-6G/Ly-6C (Gr-1)-AlexaFluor 700 (RB6-8C5) BioLegend cat#: 108422 (dilution: 1/200)
anti-Ly-6C-PECy7 (clone: AL-21) BD Biosciences cat#: 560593 (dilution: 1/200)
anti-Ly-6G-AlexaFluor 700 (clone: 1A8) BD Biosciences cat#: 561236 (dilution: 1/200)
anti-TER119-AlexaFluor 700 (clone: TER-119) BioLegend cat#: 116220 (dilution: 1/200)
anti-F4/80-Biotin (clone: BM8) eBioscience cat#: 13-4801-81 (dilution: 1/600)
anti-I-A/I-E-BV605 (clone: M5/114.15.2) BD Biosciences cat#: 563413 (dilution: 1/600)
anti-Siglec-F-APC-R700 (clone: E50-2440) BD Biosciences cat#: 565183 (dilution: 1/200)
anti-TIM-4-PE (clone: 54(RMT4-54)) eBioscience cat#: 12-5866-80 (dilution: 1/200)
anti-TCR-AlexaFluor (clone: H57-597) BioLegend cat#: 109224 (dilution: 1/100)
anti-TCR-PECF594 (clone: H57-597) BD Biosciences cat#: 562841 (dilution: 1/200)
anti-TCR-Biotin (clone: GL3) BioLegend cat#: 118103 (dilution: 1/250)
anti-TCR-BV421 (clone: GL3) BioLegend cat#: 118119 (dilution: 1/200)
anti-NK1.1-AlexaFluor 700 (clone: PK136) BioLegend cat#: 108730 (dilution: 1/200)
anti-NK1.1-BV785 (clone: PK136) BioLegend cat#: 108749 (dilution: 1/200)
anti-NKp46-BV786 (clone: 29A1.4) BD Biosciences cat#: 741029 (dilution: 1/200)
anti-FoxP3-APC (clone: FJK-16s) eBioscience cat#: 17-5773-80 (dilution: 1/200)
anti-FoxP3-PEeFluor610 (clone: FJK-16s) eBioscience cat#: 61-5773-82 (dilution: 1/200)
anti-RORt-PE (clone: B2D) eBioscience cat#: 12-6981-80 (dilution: 1/200)
anti-RORt-PE-eFluor610 (clone: B2D) eBioscience cat#: 61-6981-80 (dilution: 1/200)
anti-T-bet-PECy7 (clone: 4B10) eBioscience cat#: 25-5825-81 (dilution: 1/200)
anti-Ki67-BV786 (clone: B56) BD Biosciences cat#: 563756 (dilution: 1/100)
anti-IL-10-PE (clone: JES5-16E3) eBioscience cat#: 12-7101-82 (dilution: 1/200)
anti-IL-17A-AlexaFluor 488 (clone: TC11-18H10.1) BioLegend cat#: 506909 (dilution: 1/200)
anti-IL-22-PerCP-eFluor710 (clone: IL22JOP) eBioscience cat#: 46-7222-80 (dilution: 1/100)
anti-IFN-BV605 (clone: XMG1.2) BioLegend cat#: 505839 (dilution: 1/200)
Streptavidin-PE-CF594 BD Biosciences cat#: 562284 (dilution: 1/500)
Streptavidin-BV421 BioLegend cat#: 405226 (dilution: 1/250)

Validation | All antibodies came from commercial vendors and were validated by the manufacturers on their official website.

Animals and other organisms

Policy information about studies involving animals; ARRIVE guidelines recommended for reporting animal research

Laboratory animals	6-7-week-old male C57BL/6JBomTac, C57BL/6JRj, C57BL/6N, C57BL/6N-Rag2Tm1/CipheRj (RAG2-/-) mice were acquired from Taconic Laboratories, Denmark, or Janvier Labs, France, respectively, as detailed in the manuscript.
Wild animals	This study did not involve wild animals.
Field-collected samples	This study did not involve samples collected from the field.
Ethics oversight	All experiments were conducted in accordance with the EU directive 2010/63/EU as approved by the Danish Animal Experiments Inspectorate (#2014-15-2934-01,027).

Note that full information on the approval of the study protocol must also be provided in the manuscript.

Flow Cytometry

Plots

Confirm that:

- [x] The axis labels state the marker and fluorochrome used (e.g. CD4-FITC).
- [x] The axis scales are clearly visible. Include numbers along axes only for bottom left plot of group (a 'group' is an analysis of identical markers).
- [x] All plots are contour plots with outliers or pseudocolor plots.
- [x] A numerical value for number of cells or percentage (with statistics) is provided.

Methodology

Sample preparation

Isolation of small intestine (SI) and large intestine (LI) lamina propria (LP) cells
Feces and mucus from LI were scraped off while SI was flushed with 1x HBSS (Gibco) containing 15 mM HEPES (Thermo Fisher Scientific). Peyer´s patches were carefully excised from SI. SI and LI were opened longitudinally, cut into approx. 1 cm pieces and washed 3 times with 1x HBSS (Gibco) containing 15 mM HEPES (Thermo Fisher Scientific), 5% FCS (ViralexTM, PAA Laboratories), 50 µg/mL gentamycin (Gibco) and 2 mM EDTA (Invitrogen, Life Technologies) for 15 min at 37 ˚C. During the first incubation step, 0.15 mg/mL DL-dithiothreitol (Sigma-Aldrich) was added to LI samples. LI, but not SI samples, were shaken on an orbital shaker at 350 rpm during incubation. After each incubation step, SI, but not LI samples, were shaken by hand for 10 s. Media containing epithelial cells and debris were discarded by filtration through a 250 µm mesh (Tekniska Precisionsfilter, JR AB). The remaining tissue was digested for 20-28 min at 37 ˚C under magnetic stirring (350-500 rpm) in R-10 media (RPMI 1640 (Gibco) with 10% FCS (Viralex, PAA Laboratories), 10 mM HEPES (Thermo Fisher Scientific), 100 U/mL penicillin (Gibco), 100 µg/mL streptomycin (Gibco), 50 µg/mL gentamycin (Gibco), 50 µM 2-mercaptoethanol (Gibco) and 1 mM sodium pyruvate (Gibco)) containing 1 mg/ml collagenase P (Roche) and 0.03 mg/mL DNase I (Roche). After digestion, SI-LP and LI-LP cells were purified by density gradient centrifugation (600 rcf for 20 min at 22 ˚C, acceleration 5 and brake 0) with 40/70 % Percoll (GE Healthcare). Cell suspensions were subsequently filtered through 100 µm cell strainers (BD Biosciences) and re-stimulated in vitro prior to staining for flow cytometric analysis.

Isolation of liver cells
To isolate liver cells, livers were collected from 1x PBS (Gibco)-perfused mice, cut into small pieces and digested for 40 min at 37 ˚C in R-10 media (RPMI 1640 (Gibco) with 10% FCS (Viralex, PAA Laboratories), 10 mM HEPES (Thermo Fisher Scientific), 100 U/mL penicillin (Gibco), 100 µg/mL streptomycin (Gibco), 50 µg/mL gentamycin (Gibco), 50 µM 2-mercaptoethanol (Gibco) and 1 mM sodium pyruvate (Gibco)) containing 0.3 mg/ml collagenase IV (Roche), 0.21 mg/mL collagenase D (Roche) and 0.05 mg/mL DNase I (Roche) on an orbital shaker (370 rpm). After digestion, liver cells were filtered through 100 µm cell strainers (BD Biosciences) and subsequently purified by density gradient centrifugation (800 rcf for 20 min at 22 ˚C, acceleration 5 and brake 0) with 40/70 % Percoll (GE Healthcare). Cell suspensions were either re-stimulated in vitro prior to or directly used for flow cytometric analysis.

Ex vivo stimulation of SI-LP, LI-LP and liver cells
SI-LP, LI-LP and liver cells were re-stimulated in vitro as previously described27. Briefly, cells were simulated in R-10 media in the presence of either 20 ng/mL IL-23 (R&D Systems) or 250 ng/mL PMA (Sigma-Aldrich) in combination with 0.5 µg/mL ionomycin (Sigma-Aldrich) for 4 hrs at 37 ˚C. After 1 hr incubation, 10 µg/mL brefeldin A (BioLegend) was added.

Flow cytometry
Flow cytometry was performed according to standard procedures. Cell aggregates (identified in FSC-H or FSC-W vs. FSC-A scatter plots) and dead cells identified by using Zombie Aqua or Zombie UV Fixable Viability Kit (BioLegend) were excluded from analyses. Intracellular staining was performed using the eBioscience FoxP3/Transcription Factor Staining Buffer Set (eBioscience) according to the manufacturer’s instructions.

Instrument | Data was acquired on a LSRII (BD Biosciences).

Software	FlowJo software (Tree Star).
Cell population abundance	N/A: We did not sort any cells for subsequent transfer and thus have not confirmed the purity of such populations.
Gating strategy	Cell aggregates (identified in FSC-H or FSC-W vs. FSC-A scatter plots) and dead cells identified by using Zombie Aqua or Zombie UV Fixable Viability Kit (BioLegend) were excluded from analyses. Specific gating strategies are presented in the manuscript as relevant supplementary figures.

☒ Tick this box to confirm that a figure exemplifying the gating strategy is provided in the Supplementary Information.
